# Vaccination with staphylococcal protein A protects mice against systemic complications of skin infection recurrences

**DOI:** 10.3389/fimmu.2024.1355764

**Published:** 2024-03-11

**Authors:** Andrea Paola Mandelli, Greta Magri, Marco Tortoli, Stefania Torricelli, Donatello Laera, Fabio Bagnoli, Oretta Finco, Giuliano Bensi, Michela Brazzoli, Emiliano Chiarot

**Affiliations:** ^1^ Bacterial Vx Unit, GlaxoSmithKline, Siena, Italy; ^2^ Animal Resource Center, GlaxoSmithKline, Siena, Italy; ^3^ TRD, GlaxoSmithKline, Siena, Italy; ^4^ Infectious Disease Research Unit, GlaxoSmithKline, Upper Providence, PA, United States

**Keywords:** immunomodulators, SSTIs, mouse model, *Staphylococcus*, bacteremia

## Abstract

Skin and soft tissue infections (SSTIs) are the most common diseases caused by *Staphylococcus aureus* (*S. aureus*), which can progress to threatening conditions due to recurrences and systemic complications. Staphylococcal protein A (SpA) is an immunomodulator antigen of *S. aureus*, which allows bacterial evasion from the immune system by interfering with different types of immune responses to pathogen antigens. Immunization with SpA could potentially unmask the pathogen to the immune system, leading to the production of antibodies that can protect from a second encounter with *S. aureus*, as it occurs in skin infection recurrences. Here, we describe a study in which mice are immunized with a mutated form of SpA mixed with the Adjuvant System 01 (SpA_mut_/AS01) before a primary *S. aureus* skin infection. Although mice are not protected from the infection under these conditions, they are able to mount a broader pathogen-specific functional immune response that results in protection against systemic dissemination of bacteria following an *S. aureus* second infection (recurrence). We show that this “hidden effect” of SpA can be partially explained by higher functionality of induced anti-SpA antibodies, which promotes better phagocytic activity. Moreover, a broader and stronger humoral response is elicited against several *S. aureus* antigens that during an infection are masked by SpA activity, which could prevent *S. aureus* spreading from the skin through the blood.

## Introduction

*Staphylococcus aureus* (*S. aureus*) is a commensal human pathogen that persistently colonizes the skin and nares, as well as the gastrointestinal tract, of about one-third of the human population (1), and colonization is considered a key risk factor for infection (2). *S. aureus* is responsible for a wide variety of diseases, ranging from mild and common conditions to life-threatening diseases mainly thanks to its broad spectrum of virulence factors that can contribute to tissue colonization, infiltration, tissue damage, and distant diseases (3). Skin and soft tissue infections (SSTIs) are the most common *S. aureus*-mediated diseases. Uncomplicated SSTIs affect millions of patients of all ages worldwide every year (4–6), and the development of serious complications as systemic disseminations, even if relatively rare, can radically change the course of infection especially when caused by antibiotic-resistant strains (7). The capability of *S. aureus* to establish such a different kind of infection is mainly due to its plethora of virulence factors. Among these, staphylococcal protein A (SpA) plays a determinant role in establishing persistent and often recurrent infections, avoiding bacterial recognition and elimination by the host immune system. SpA, in fact, allows bacterial survival by exploiting two distinct antibody binding activities through its binding domains: (i) binding to immunoglobulins (in humans, all but IgD, IgE, and IgG3) through the Fcγ domain (8, 9), preventing opsonophagocytosis by sequestering antibodies and displaying them on the bacterial surface in an incorrect orientation (10–12); and (ii) binding to the VH3 domain of IgM/IgD present on the surface of B lymphocytes, triggering superantigen activity and causing cell necrosis (13–15). One of the main roles of the SpA is therefore to facilitate bacterial survival and to allow *S. aureus* to escape the host immune system. Previous works have shown that a nontoxigenic version of SpA (denominated SpA_KKAA_, hereby indicated as SpA_mut_), engineered by replacing essential amino acid residues in the binding domain, was able to elicit polyclonal antibodies that promote opsonophagocytosis and killing (OPK) of staphylococci and suppress their B-cell superantigen activity (11). Mouse monoclonal antibodies specific for SpA_mut_ also neutralize SpA and promote OPK of *S. aureus*, together with the development of antibody responses against many different antigens (16). Furthermore, a previous work has shown that immunization with the non-toxic version of the SpA was able to protect guinea pigs from *S. aureus*-mediated systemic infection and to elicit a broader humoral response in animals vaccinated before infection as compared to only infected animals (8). Considering these observations, the development of a broader immune response after immunization with SpA and infection with *S. aureus* could represent an effective protection against a second exposure to the pathogen, as in recurrent skin infections. To understand if the immunomodulator properties of SpA could affect the humoral response after an *S. aureus* skin infection, we used a mouse model of skin infection and recurrence and characterized the humoral response in animals vaccinated with SpA_mut_.

## Methods

### Bacterial strains and preparation of inoculum for infection

*S. aureus* USA300 LAC strain was used for the *in vivo* model of skin infection and recurrence. Bacteria were grown in tryptic soy broth (TSB) at 37°C in agitation until the early exponential phase is reached and diluted 1:1 in a freezing solution, composed of phosphate-buffered saline (PBS) + 10% bovine serum albumin (BSA) + 10% L-glutamic acid. Aliquots were conserved at −80°C in cryovials until use.

For the inoculum preparation, 2-mL frozen stocks of bacteria were thawed and diluted in 48 mL of fresh TSB and incubated at 37°C at 250 rpm until bacteria reached the early exponential phase (OD_600 _= 2.0), at approximately 1.5–2 × 10^9^ colony-forming units (CFUs)/mL. Bacteria were then washed once in sterile PBS and diluted with sterile PBS to obtain 4 × 10^8^ CFUs/mL or 8 × 10^8^ CFUs/mL for the skin infection or recurrence models, respectively.

*S. aureus* USA300 LAC strain expressing the green fluorescent protein (GFP) was used for the internalization assays. Bacteria were streaked on a tryptic soy agar (TSA) plate and incubated overnight (O/N) at 37°C + 5% CO_2_. The following day, pre-inocula were prepared from single colonies and they were incubated O/N at 37°C at 250 rpm. The following day, the cultures were diluted in fresh TSB and incubated at 37°C and 250 rpm for approximately 3 h, until they reached the early exponential phase (OD_600 _= 2). Bacteria were centrifuged at 2,400 rpm and 4°C for 10 min and washed twice with RPMI 1640 GlutaMAX + 25 mM HEPES + 0.05% BSA. After the final resuspension, the OD_600_ of bacteria was measured and adjusted to an OD_600_ of 1.0. Bacteria were aliquoted in 2-mL cryovials and stored at −20°C until use.

### Animal care and ethical statements

Animal husbandry and experimental procedures were ethically reviewed and carried out in accordance with European Directive 2010/63/EU, Italian Decree 26/2014, and GSK Vaccines’ Policy on the Care, Welfare and Treatment of Animals, and were approved by the Italian Ministry of Health (authorization 123/2015-PR). Animals were kept in an AAALAC-accredited facility. Upon arrival, animals were randomly distributed in different experimental groups in individually ventilated cages (IVC, Sealsafe Plus GM500 by Tecniplast). The acclimation lasted for a period of 5 days. At the end of the acclimation period, each animal was identified by an individual tattoo. All animals had *ad libitum* access to GMP-grade food (Mucedola 4RF25 TOP CERTIFICATE) and bottled, filtered, tap water. Certified, irradiated cellulose bags containing Mucedola SCOBIS UNO bedding and carboard tunnels (ANTRUM) or plexiglass mouse houses were provided within the cages. A few food pellets in the cage were also used as enrichment for foraging and additional gnawing. Cage and bedding changes were performed once every 2 weeks in agreement with the cage supplier’s indications. Air supplied in IVC was 100% fresh air filtered by an EPA filter via the IVC system, with 60–75 air changes per hour. The animal room conditions were as follows: temperature, 21°C (± 3°C); relative humidity, 50% (range 30%–70%); and 12-h light/12-h dark cycle. Pressure, temperature, and relative humidity were recorded continuously by room probes, while the IVC system recorded the individual motors’ performance. The light cycle setting was ensured by a validated alarm system.

### Mouse model of skin infection and recurrence

The mouse model of skin infection and recurrence was performed as depicted in the Supplementary Materials (see **Supplementary Material Figure S1**). Five-week-old female specific pathogen-free (SPF) C57BL/6 mice were immunized twice intramuscularly (IM), 28 days apart. Animals were immunized with 10 µg of a SpA_mut_ adjuvanted with AS01, in a final volume of 50 µL, 25 µL/posterior leg. The control group (sham) was immunized with the formulation buffer alone (10 mM Na_2_HPO_4_ and 150 mM NaCl, pH 6.1).

For the *in vivo* vaccine efficacy studies, 4 weeks after the second immunization, the back of mice was shaved under anesthesia (3.5% isoflurane) using an electric razor and depilatory cream. For the primary skin infection, the day after shaving, mice were inoculated subcutaneously (SC) in the right flank with 2 × 10^7^ CFU/50 µL of *S. aureus* USA300 LAC strain, under anesthesia (3.5% isoflurane). Six weeks after, mice were shaved and infected again as previously described in the opposite flank with 4 × 10^7^ CFU/50 µL of *S. aureus* USA300 LAC strain to establish the model of skin infection recurrence.

Mice were observed daily for clinical signs of disease up to 14 days after infection. Mice were scored on the basis of pre-established parameters, and they were euthanized in case they exhibited pre-established humane endpoints in agreement with local Animal Welfare Policies (see **Supplementary Materials Table 1**). Dermonecrotic lesions were photographed and measured daily from day 4 to day 7 post-infection. At specific time points (which are shown in **Supplementary Figure 1**), blood was withdrawn from mice and serum was collected by centrifugation, for future serological analysis. At determined time points, mice were euthanized, and skin biopsies and kidneys were collected, homogenized, serially diluted and plated for bacterial burden, and expressed as CFU/mL for skin biopsies or as dissemination index for kidneys.

### Coupling of magnetic beads with SpA_WT_ and SpA_mut_

For all the Luminex-based assays, 20 µg of recombinant SpA_WT_ or SpA_mut_ was chemically coupled to 1.25 × 10^6^ MagPlex beads (Luminex Corporation), through an automated coupling method with a liquid handling workstation (Hamilton – Microlab STAR IVD). Briefly, antigens were coupled by a two-step carbodiimide procedure during which microsphere carboxyl groups are first activated with 1-ethyl-3-(3-dimethylaminopropryl) carbodiimide hydrochloride (EDC, Pierce), in the presence of sulfo-NHS (Pierce) to form a sulfo-NHS-ester intermediate. The reactive intermediate is then replaced by a reaction with the primary amine of the target molecule to form a covalent amide bond.

### Serological analysis

Total SpA_mut_-specific IgG titers were evaluated with an automatized Luminex assay, incubating serially diluted mice sera with SpA_mut_-coupled beads for 1 h with vigorous shaking. After washing, IgG titers were detected using an R-Phycoerythrin AffiniPure F(ab′)_2_ Goat anti-mouse IgG (Jackson ImmunoResearch Labs, Cat #115-116-072, RRID: AB_2338627) through a FLEXMAP 3D reader (Luminex Corporation). Median fluorescence intensities (MFIs) of the samples, subtracted to the MFI of the blanks (MFI-Bkgd), were interpolated to a standard curve, expressing the IgG titer as relative light units per milliliter (RLU/mL). For each sample tested, the experiment was considered valid if a titer was observed in at least three points of the dilution and if these titers were interpolated in the linear portion of the standard curve; the final IgG titer of each value was expressed as the median value of the observed titers.

For the IgG subclass analysis, threefold serially diluted pools of sera collected after the second dose of immunization (D52, post II) or after skin infection (D72, 2 weeks post-infection) were incubated with SpA_mut_-coupled beads for 1 h with vigorous shaking, repeating the assay in five different 96-well plates. After washing, the specific IgG subclass was detected using the appropriate R-Phycoerythrin AffiniPure F(ab′)_2_ Goat anti-mouse IgG (Cat #115-116-072, RRID : AB_2338627), IgG1 (Cat #115-115-205, RRID : AB_2338620), IgG2a (Cat # 115-115-206, RRID : AB_2338621), IgG2b (Cat #115-115-207, RRID : AB_2338622), or IgG3 (Cat #115-115-209, RRID : AB_2338624) secondary antibody (Jackson ImmunoResearch Labs). Results were detected using a Luminex 200 instrument system (Luminex Corporation), and they were reported as the ratio between the RLU/mL of the different IgG subclasses.

For the characterization of the avidity, a mouse standard serum and mice sera obtained after immunization (D52, post II) and after skin infection (D72, 2 weeks post-infection) were threefold serially diluted and incubated in duplicate with SpA_mut_-coupled beads for 1 h with vigorous shaking. After washing, one-half of the samples was incubated with vigorous shaking either with 2 M ammonium thiocyanate or with PBS for 30 min. After washing, the remaining IgGs were detected with an R-Phycoerythrin AffiniPure F(ab′)2 Goat anti-mouse IgG (Jackson ImmunoResearch Labs) using a Luminex 200 reader (Luminex corporation). Results were reported as avidity percentage, calculated on the basis of the ratio of the antibody titers recovered after incubation with ammonium thiocyanate with respect to titers observed after incubation with PBS.

### Luminex displacement assay

The displacement assay was performed as described elsewhere (17). Briefly, SpA_WT_-coupled beads (prepared as already described above) were saturated with 10 µg/mL of biotinylated-human IgGs for 30 min under vigorous shaking. After washing, saturated beads were incubated with threefold serially diluted total IgGs purified from mice sera collected after immunization (D52, post II), after skin infection (D72, 2 weeks post-infection) or with commercial total mouse IgGs as negative control (ChromPure Mouse IgG, ImmunoResearch Labs, Cat #015-000-003, RRID : AB_2337188), for 30 min with shaking. After washing, the amount of biotinylated-human IgGs bound to the SpA_WT_-coupled beads was detected using 10 µg/mL of Streptavidin, R-Phycoerythrin Conjugate (SA-PE), through a FLEXMAP 3D reader. Results are shown as the percentage of antibody displaced, normalized on the signal obtained without adding mouse IgGs.

### Staphylococcal protein microarray

The staphylococcal protein microarray was generated by spotting relevant *S. aureus* antigens that were previously selected, expressed in competent *E. coli* cells, purified, and characterized as described elsewhere (18–20). Briefly, staphylococcal antigens were spotted randomly in quadruplicate on ultra-thin nitrocellulose-coated glass slides (Maine Manufacturing) using an ink-jet spotter (Arrayjet) in a cabinet at 18°C and 40% humidity (see **Supplementary Table S2** for the complete list of spotted antigens). Slides were conserved in the dark at 4°C until use. After saturating the slides with a blocking buffer (BlockIt, Arraylt), diluted sera samples (*n* = 9) obtained 2 weeks after infection from immunized (SpA_mut_/AS01 and infected) or non-immunized (sham and infected) mice were incubated on slides for 1 h in the dark in a humid chamber. After washing, signals were detected with an Alexa Flour 647 anti-mouse IgG secondary antibody (Jackson ImmunoResearch Labs, Cat #115-605-006, RRID : AB_2338903) and slides were rinsed and left to dry. Slides were scanned using an InnoScan 710 AL (INNOPSYS) and images were generated by the Mapix Microarray Image Analysis Software. Fluorescence intensities of spots were determined using ImaGene 9.0 software (Biodiscovery Inc.) and data analysis was performed using R scripts.

Each spotted antigen was considered as “positive” if recognized by the majority of tested sera (at least five out of nine).

### Opsonophagocytosis assay

THP-1 cells (ATCC TIB-202, RRID: CVCL_0006) were cultured in RPMI 1640 medium supplemented with GlutaMAX and HEPES (Gibco), 10% FBS, and 5 μg/mL of penicillin/streptomycin, according to manufacturer indications. Single sera collected from animal studies were heat-inactivated at 56°C for 30 min. The opsonophagocytosis assay was performed as described elsewhere (17). Briefly, 3–4 × 10^6^ CFUs/mL of *S. aureus* USA300 GFP strain were pre-opsonized with mouse sera obtained from normal mice (Pre-immune), immunized mice (SpA_mut_/AS01 post II), 2 weeks after infection from non-immunized mice (sham and infected), and immunized mice (SpA_mut_/AS01 and infected) for 30 min at 37°C under vigorous shaking. A total of 3–4 × 10^4^ THP-1 cells were added to allow opsonization of bacteria, and incubation continued for an additional 30 min (bacteria-to-cell ratio of 200:1). After adding lysostaphin (Sigma-Aldrich) to lyse non-internalized bacteria, bacteria and cells were fixed with 2% formaldehyde for 1 h on ice prior to acquisition at FACS CANTO II (Becton Dickinson) and data collected were analyzed using the FACSDiva 8.0.1 software. Gating strategy was as follows: (i) THP-1 cells; (ii) singlet cells; and (iii) FITC-A, representative of cells that have effectively internalized GFP-expressing bacteria. The median percentages of GFP^+^ THP-1 cells were normalized on the median signal obtained with pre-immune sera, and results were then represented as fold change with respect to the median of pre-immune sera.

### Statistical analysis

**Figure 1:**
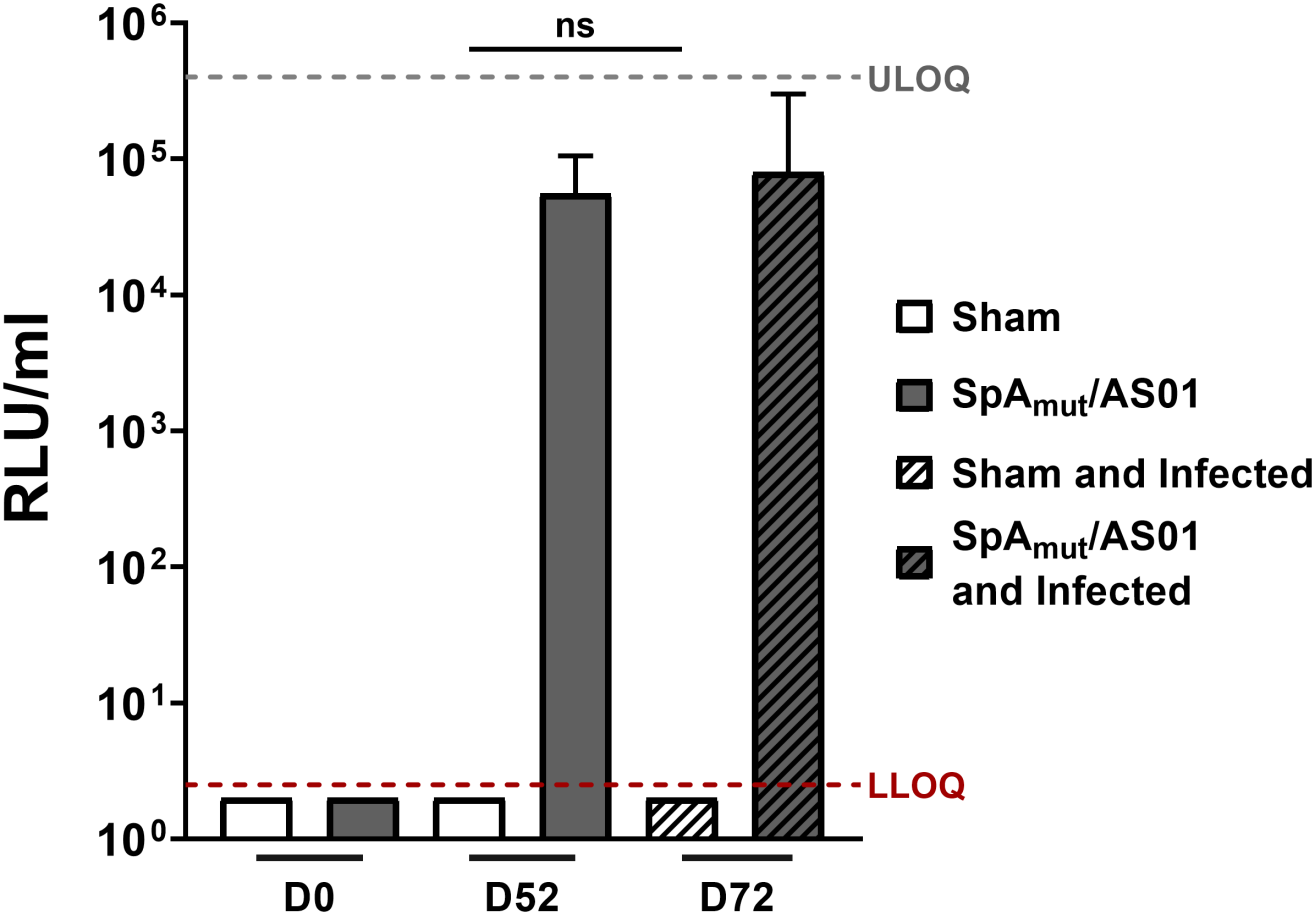
Subcutaneous infection with *S. aureus* does not induce anti-SpA_mut_IgG in mice. Anti-SpA_mut_ IgG titration expressed as relative light units (RLU) per milliliter in sera of animals only immunized, only infected,or immunized and infected. Pre-immune (Day 0, D0), post II (D52), or post-infection (D72) sera were analyzed and titers against SpA_mut_ were reported in the graph. Sham animals received only buffer, while SpA_mut_/AS01 animals were immunized with the proposed vaccine. The median value and the 95% confidence interval were reported for each group and condition. Groups were represented by at least 7 animals (range, 7–11 animals/group). LLOQ, lower limit of quantification; ULOQ, upper limit of quantification. n.s. *p*_val_ > 0.05. The Kruskal–Wallis and uncorrected Dunn’s posttest was used to assess significance.

**Figure 2:**
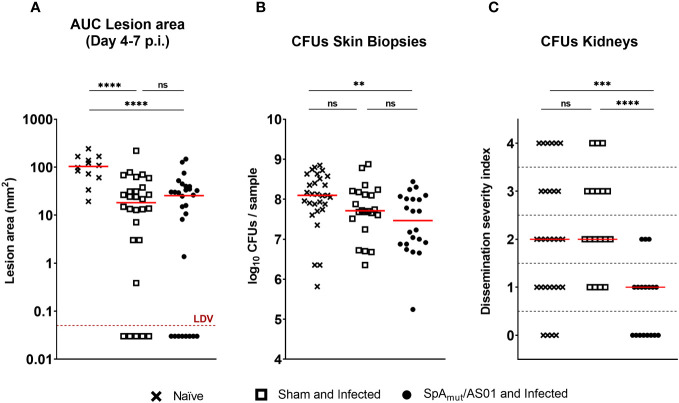
Vaccination with SpA_mut_/AS01 protects against local manifestations and systemic *sequelae* in a skin infection recurrence mouse model of *S. aureus* infection. Skin **(A, B)** and systemic **(C)** readouts used to assess protection of SpA_mut_/AS01 vaccine in the skin infection recurrence model. **(A)** Area under the curve (AUC) indicated as mm2 of dermonecrotic lesion developed from day 4 to day 7 after skin infection recurrence. The red dotted line represents the Lower Detectable Value (LDV). ****p_val_< 0.0001. **(B)** Log10 of colony-forming unit (CFU) counts enumerated in homogenized skin biopsies collected 7 days after skin infection recurrence. **p_val_< 0.01; n.s. *p*_val_ > 0.05. **(C)** Dissemination severity index assigned to single mice based on CFU counts recovered in the kidneys of infected mice 7 days after skin infection recurrence. Score 0, no bacteria; Score 1, very low dissemination (1–10 CFU/kidneys); Score 2, minimal dissemination (11–100 CFU/kidneys); Score 3, mild dissemination (101–1,000 CFU/kidneys); Score 4 severe dissemination (>1,001 CFU/kidneys). ***p_val_< 0.001; ****p_val_< 0.0001; n.s. *p*_val_ > 0.05. For all the graphs above, each single dot represented data from a single animal, and red lines were median values of the groups. A total of 29 animals belong to the age-paired naïve mice (Naïve) group, 18 animals make up the group of mice not vaccinated but exposed to *S. aureus* during the first infection (sham and infected), and 18 animals belong to the group of mice vaccinated and infected (SpA_mut_/AS01 and Infected). The Kruskal–Wallis and uncorrected Dunn’s post-test was used to assess significance among groups.

**Figure 3:**
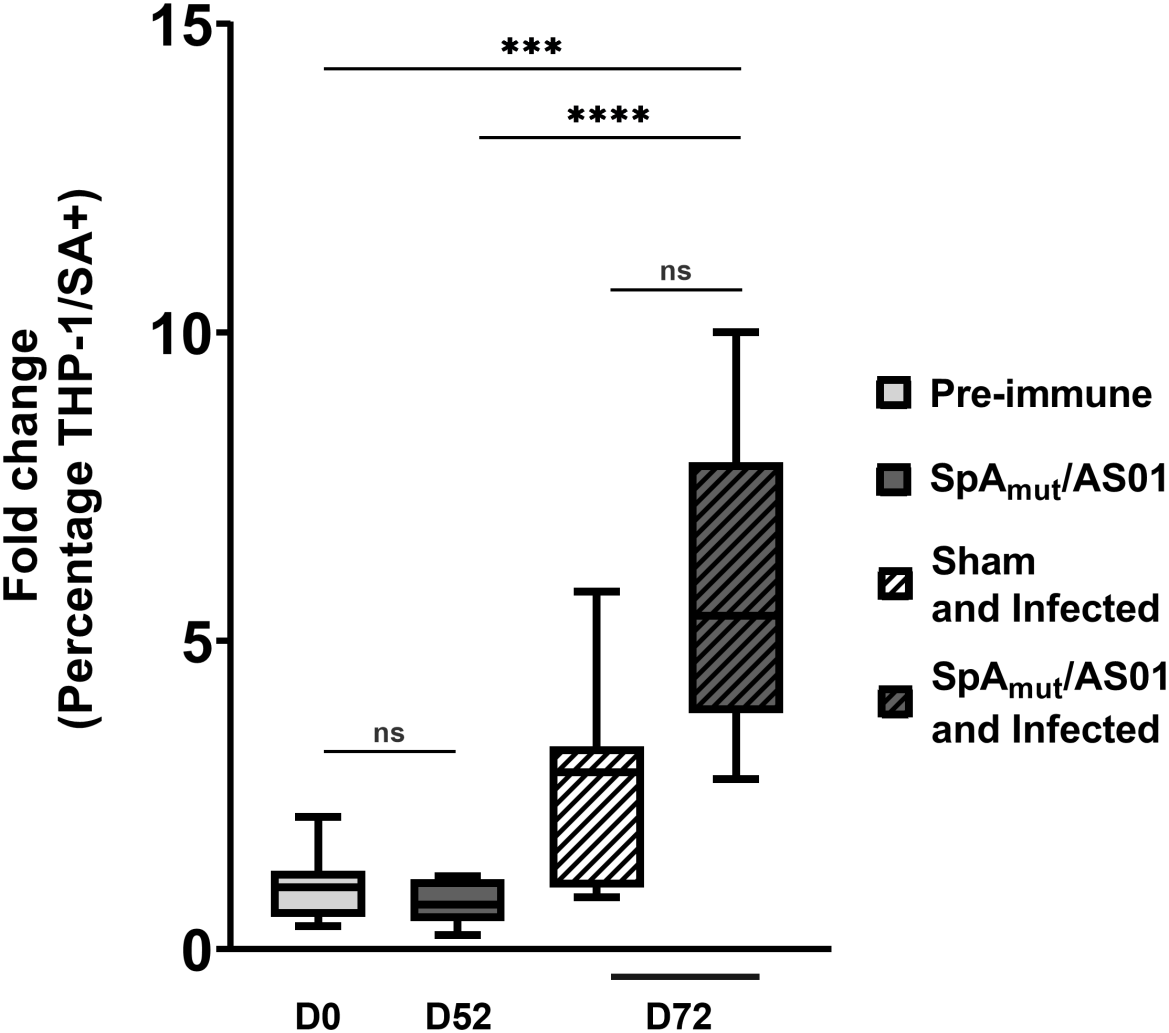
Antibodies in sera from SpA_mut_/AS01 and infected mice increase *S. aureus* phagocytosis by specialized human cells. Percentage of human THP-1 monocytic cells that internalized GFP-labeled *S. aureus* USA300 LAC strain *in vitro*. Box-and-whiskers representation of THP-1 cells that have internalized bacteria. Lines delimiting the boxes reported the median value of the group with 25 and 75 percentiles. The whiskers reported 10 and 90 percentiles. Eight pools (from at least five mouse sera for each group) were used for the assay. ***p_val_< 0.001; ****p_val_< 0.0001; n.s. *p*_val_ > 0.05. The Kruskal–Wallis and uncorrected Dunn’s post-test was used to assess significance.

**Figure 4:**
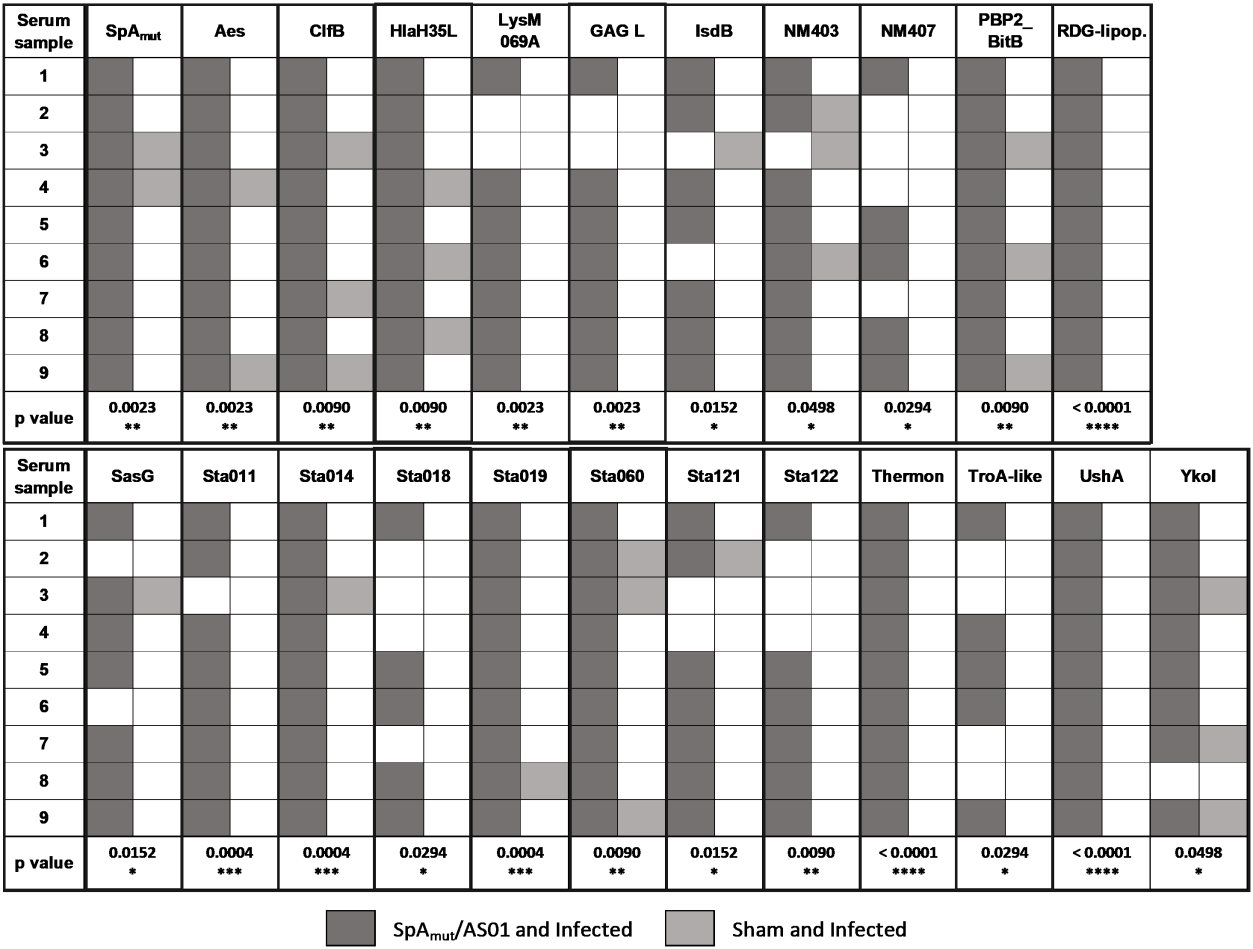
Vaccination with SpA_mut_/AS01 expands the antibody repertoire following an *S. aureus* infection. Nine single sera obtained from immunized and infected (SpA_mut_/AS01 and infected) and from infected-only animals (sham and infected) were assessed against the staphylococcal protein microarray. Out of the 96 antigens spotted on the array, 23 reacted with higher frequency with SpA_mut_/AS01 and infected sera, with respect to sera obtained from infected-only animals. Filled box: reactive serum; non-filled box: non-reactive serum. GAG L: Glycosaminoglycan lyase; RDG-lipop.: RDG lipoprotein; Thermon: thermonuclease. *p_val_< 0.05; **p_val_< 0.01; ***p_val_< 0.001; ****p_val_< 0.0001. Fisher’s exact *t-*test was used to evaluate significant differences between recognition frequencies for each antigen.

Statistical analysis was performed using the GraphPad Prism Software version 8.0.0. The non-parametric Mann–Whitney *U*-test was used to assess differences between two groups, as for the evaluation of IgG titers against SpA_mut_ (**Figure 1**). The one-way ANOVA Kruskal–Wallis and uncorrected Dunn’s post-test was used to assess differences between three or more groups, as in the *in vivo* results or in the internalization assay (**Figures 2A, B**, **3**). Finally, the Fisher’s exact *t*-test was used to assess differences between frequencies, as in the dissemination severity index or the protein microarray results (**Figures 2C**, **4**). A *p* (value) ≤ 0.05 was considered significant for all these analyses. Legend: *p ≤ 0.05; **p ≤ 0.01; ***p ≤ 0.001; ****p ≤ 0.0001; ns = not significant, *p* > 0.05.

The area under the curve (AUC) was used to perform a cumulative data analysis on the dermonecrotic lesion areas (**Figure 2A**). The trapezoidal AUC of the dermonecrotic lesion areas, for a single day, was calculated using the following formula:


$${\text{AUC}}_{0-1}=\;\frac{\left({\text{B}}_{\text{t}0}+{\text{b}}_{\text{t}1}\right)\text{ x }\left({\text{t}}_{0}-{\text{t}}_{1}\right)}{2}$$

where *B* stands for major base, *b* denotes minor base, and *t* stands for time point.

The AUC was calculated for each time point after infection in the single animal and eventually summed together to have a final AUC value representing the whole time period after the infection.

## Results

### Immunization with SpA_mut_/AS01 protects mice against systemic *sequelae* in a model of *Staphylococcus aureus* skin infection and recurrences

Vaccination with SpA_mut_ was shown to protect guinea pigs against systemic disease and to enable the host to develop a broader antibody repertoire against several staphylococcal antigens otherwise masked by SpA activity, which could protect from a second encounter with *S. aureus* (8). Given these premises, we developed an animal model to evaluate the contribution of SpA-specific antibodies in mediating the protection to systemic *sequelae* of skin infection recurrences.

To achieve this, C57BL/6 mice were immunized with SpA_mut_ adjuvanted with AS01 (SpA_mut_/AS01). Four weeks after immunization, mice were inoculated subcutaneously (SC) in the right flank with *S. aureus* USA300 LAC strain (primary infection). Six weeks after the primary infection, mice were infected again with the same bacterial strain in the opposite flank (secondary infection), mimicking a skin infection recurrence (see **Supplementary Figure 1** for the experimental design of the model). Different readouts were used to monitor the effect of vaccination and of the infection, both locally and systemically. Results were compared with those obtained with mock immunized mice, infected either once or twice.

A previous exposure to the pathogen contributed to reduce dermonecrotic lesions developed by mice during a second infection by approximately 80% (the median value of naïve mice was 103.3 mm^2^ and the median value of sham and infected mice was 18.25 mm^2^), but no additional effects due to a prior vaccination with SpA_mut_/AS01 were observed (**Figure 2A**). Mouse vaccination contributed nevertheless to significantly reduce bacterial load in the skin compared to the sham and infected mice (*p*_val_ sham and infected vs. naïve was 0.07, *p*_val_ SpA_mut_/AS01 and infected vs. naïve was 0.0057; **Figure 2B**). Notably, a pre-existing immunity present in sham and infected mice did not have any impact on the ability of *S. aureus* to migrate from the skin to the kidneys, while immunization with SpA_mut_/AS01 almost fully eradicated systemic complications during skin infection recurrence (**Figure 2C**).

We also evaluated a possible effect of immunization with the proposed vaccine in a model of skin infection but no reduction in dermonecrotic area size and CFU counts either in the skin or in the kidneys as observed (see **Supplementary Material Figure 2**).

### *S. aureus* subcutaneous infection increases the affinity of anti-SpA antibodies following vaccination with SpA_mut_/AS01

Immunization with SpA_mut_/AS01 expanded the immune response to the pathogen after infection of mice, which overall contributed to control local disease (bacterial load in skin lesions) and bacterial dissemination to kidneys in a skin infection recurrence model.

In an attempt to understand the mechanisms underlying this observation, we firstly evaluated the antibody response generated against the vaccine candidate after immunization and infection. As shown in **Figure 1**, infection after vaccination did not boost the quantity of anti-SpA_mut_ antibodies generated by vaccination itself. Interestingly, no antibodies against SpA_mut_ were found in sera from sham and infected mice, underlying once more the ability of SpA to mask itself and, potentially, also other virulence factors to the host immune system (**Figure 1**).

We then wondered whether a change in the quality of anti-SpA_mut_ IgG immune response could be detected after infection. IgG subclasses of anti-SpA_mut_ specific antibodies were therefore analyzed in mouse sera, showing that no significant changes in the antibodies repertoire occurred during infection (**Table 1**). Eventually, affinity of anti-SpA_mut_ IgG was characterized in sera collected from mice before and after subcutaneous infection. Importantly, anti-SpA_mut_ antibodies strongly increased in affinity for their target during infection, with a relative percentage of IgG bound to the protein in the presence of 2 M ammonium thiocyanate that passed from 28% to 84%.

**Table 1:** SpA_mut_-specific IgG subclasses composition analysis after immunization and infection.

Time point	Titer ratios		
	IgG1/IgG2a	IgG1/IgG3	IgG2a/IgG3
D52 (SpA_mut_/AS01 post II)	0.59	0.81	1.36
D72 (2 weeks post-infection)	0.75	1.34	1.78

### SpA_mut_/AS01 vaccination followed by *S. aureus* subcutaneous infection is associated with increased functionality and “broadness” of antibody response against the pathogen

**Figure 5:**
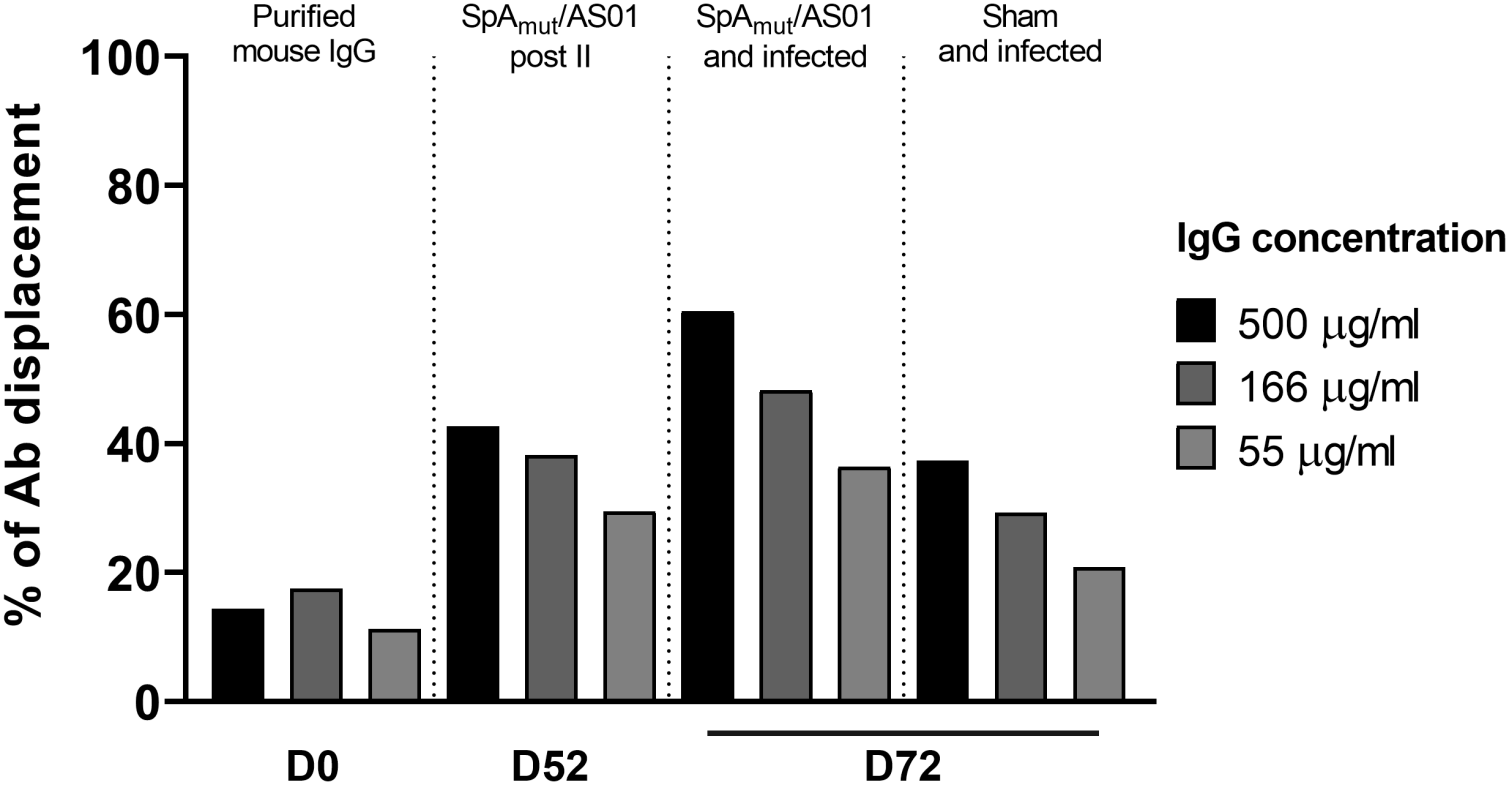
*S. aureus* infection increases the ability of vaccination to induce antibodies that allow *in vitro* human IgG displacement from SpA wild-type protein. Percentage of human IgG displaced from SpA_WT_-coupled beads by purified IgGs from sera of animals only immunized with SpA_mut_/AS01 or immunized and infected animals. Results were expressed as the percentage of human antibody displaced from the SpA_WT_, normalizing on the background signal.

The observed increased affinity might lead to more functional antibodies. To verify this hypothesis, the functionality of SpA-specific antibodies was evaluated in an *in vitro* displacement assay that measured the ability of anti-SpA IgG to displace human immunoglobulins bound to the wild-type SpA protein mainly through the Fc region (SpA_WT_). **Figure 5** shows that immunoglobulins purified from vaccinated-only or infected-only mice sera displaced human IgG bound to the wild-type protein (40% at 500 µg/mL of purified IgGs) with a twofold increase as compared to the negative control group (almost 20% of background displacement). This effect was strongly enhanced when IgG purified from animals immunized and then infected were used with a 60% IgG displacement and an increase of threefold as compared to the negative control.

Moreover, immunization with SpA derivatives followed by *S. aureus* infection was previously shown to increase antibody response against several other *S. aureus* virulence factors (1, 8). Sera from sham and infected (only infected animals) and SpA_mut_/AS01 and infected mice (animals immunized with SpA_mut_/AS01 before subcutaneous *S. aureus* infection) were therefore compared for their ability to recognize *S. aureus* antigens on a protein chip where up to 96 antigens were spotted (see **Supplementary Material Table 2** for the complete list of antigens). Among these 96 virulence factors, 23 reacted with higher frequencies when sera from SpA_mut_/AS01 immunized and infected mice were used as compared to sera from sham and infected animals. Results with the 23 antigens are shown in **Figure 4** and **Table 2**.

**Table 2:** List of the 23 antigens that reacted with higher frequency with sera from immunized and infected mice.

Protein name	Description	Localization	References	
			Known virulence factor	Preclinical data of protection
Aes	Acetyl esterase/lipase	Cytoplasmic		
Hla	α-Hemolysin toxin, cytotoxic	Extracellular	Tang et al., 2019 (21)	Adhikari et al., 2016 (22); Tran et al., 2020 (23)
LysM069A	Fragment of autolysin, involved in biofilm formation	Cell wall	Porayath et al., 2018 (24); Pulia et al., 2022 (25); Zheng et al., 2022 (26).	
Glycosamino-glycan lyase	Enzyme able to lyse GAGs (e.g., hyaluronan) contributing to the invasive capacity of *S. aureus*	Extracellular	Ibberson et al., 2016 (27)	
IsdB	Hemoglobin receptor required for heme iron utilization	Cell wall	Torres et al., 2006 (28)	Tsai et al., 2022 (29)
MSCRAMM_ ClfB	Promotes colonization by binding fibrinogen, cytokeratin	Cell wall	Lacey et al., 2019 (30)	Schaffer et al., 2006 (31)
NM403	Uncharacterized antigen belonging to CSA family	Extracellular	Schluepen et al., 2013 (32)	
NM407	Uncharacterized antigen belonging to CSA family	Extracellular	Schluepen et al., 2013 (32)	
PBP2_BitB	Substrate binding domain of an iron transporter, member of PBP2 family	Cell membrane		
RDG lipoprotein	Member of OppA transport system, involved in recycling cell wall peptides	Periplasmic		
SasG	Protein involved in biofilm formation	Cell wall	Schaeffer et al., 2015 (33)	
Sta011	Uncharacterized antigen belonging to CSA family	Extracellular	Schluepen et al., 2013 (32)	
Sta014	Member of the PBP2 family, binding domain of a nitrate transporter	Periplasmic		
Sta018	Uncharacterized substrate-binding component of an ABC-type import system	Cell wall		
Sta019	Peptidoglycan hydrolase	Extracellular	Wang et al., 2022 (34)	
Sta060	Peptide ABC transporter, member of OPPA transport system	Cell wall		
SpA_mut_	Mutated form of SpA, able to bind Fab and Fc portion of antibodies	Cell wall	Kobayashi and DeLeo, 2013 (12)	Kim et al., 2015 (8)
Thermonuclease (homologous)	Regulates biofilm formation by modulating extracellular DNA release	Extracellular	Yu et al., 2021 (35)	
TroA-like transporter	TroA-like superfamily transport ferric siderophores and metal ions	Periplasmic		
Uncharacterized lipoprotein—Sta121	Uncharacterized lipoprotein belonging to CSA family	Cell membrane	Schluepen et al., 2013 (32)	
Uncharacterized lipoprotein—Sta122	Uncharacterized lipoprotein belonging to CSA family	Cell membrane	Schluepen et al., 2013 (32)	
UshA	Belonging to 5′ nuclease family	Cell wall		
YkoI	Uncharacterized membrane protein	Cell membrane		

### Sera from SpA_mut_/AS01 vaccinated and infected mice induces phagocytosis of *S. aureus* by human monocytic cells

Sera of SpA_mut_/AS01 and infected mice, as compared to sera from only vaccinated or sham and infected mice, elicited antibodies that (i) showed an increased affinity for SpA,(ii) reduced SpA ability to bind immunoglobulins (**Figure 5**), and (iii) recognized more *S. aureus* virulence factors both secreted and exposed on the bacterial surface (**Figure 4**; **Table 2**). Antibodies present in sera of these animals before the second subcutaneous infection (recurrence) could therefore facilitate the clearance of bacteria, and this might explain why spreading of *S. aureus* from skin to kidneys was highly impaired in SpA_mut_/AS01-immunized and infected animals.

To further evaluate the contribution of SpA-specific antibodies in the elimination of *S. aureus* from the blood circulation, a human monocytic cell line (THP-1) was used to evaluate bacterial internalization in the presence of sera from different groups of mice. As reported in **Figure 3**, neither vaccination of mice with SpA_mut_/AS01 (fold change equal to 0.7 with respect to the pre-immune sera, 10% percentile was 0.23, and 90% percentile was 1.18) nor bacterial infection (fold change equal to 3 with respect to the pre-immune sera, 10% percentile was 0.84, and 90% percentile was 5.8) significantly enhanced phagocytosis as compared to the negative control. Remarkably, the combination of vaccination and infection generated sera that significantly increased the phagocytic properties of THP-1 cells compared to the negative control (fold change equal to 5, 10% percentile was 2.8, and 90% percentile was 10, *p*_val_ = 0.0007) and, interestingly, also to vaccination alone (*p*_val_< 0.0001).

## Discussion

In the present study, we have demonstrated that the efficacy of a vaccine candidate, especially if it contains an immunomodulator virulence factor, could go beyond a direct effect of antibodies and cellular response against the antigen itself. A detoxified version of Staphylococcal protein A (SpA_mut_), adjuvanted with AS01, triggered the mouse immune system to mount a broader and more mature serological response against *S. aureus* during a first encounter with the pathogen, which importantly protected the animals against a second infection. Considering that skin infection and recurrences are one of the most frequent *S. aureus*-mediated disease in humans, the results obtained in an animal model somehow mimicking the human disease are particularly relevant.

Differently from mice, humans are often pre-exposed to *S. aureus*, and this can have an impact on the immune response elicited by a subsequent vaccination and on the effect mediated by SpA (“indirect” protection mediated by SpA-antibodies). Follow-up studies should be done to further address this point.

SpA is a well-known immunomodulator virulence factor of *S. aureus* that significantly contributes to make this opportunistic pathogen one of the best adapted bacteria to human hosts (36). When exposed on bacterial surface, SpA binds the Fc (Fragment crystallizable) portion of human immunoglobulins coating *S. aureus* with a kind of “host self-dress”, a mask that makes this pathogen (and its virulence factors) hidden to the host immune system (37). Additionally, when secreted, SpA binds the VH3 domain of the Fab (Fragment antigen binding) region present on IgM/IgD receptors on the surface of B lymphocytes inducing anergy and preventing the maturation of a long-lasting memory response (12).

Overall, the functionality of anti-*S. aureus* antibodies is impaired under these conditions, and human defenses against *S. aureus* are therefore often inefficient in clearing the infection.

Recurrences and bloodstream dissemination, both strictly connected to the ability of *S. aureus* to hide in the immune system, are the hallmark of SSTIs and exemplify the inability of infected hosts to establish a protective immunity (1, 38, 39).

SpA has already been tested as a vaccine candidate in animal models against *S. aureus*-mediated infections, particularly against systemic disease (8, 40, 41). In these publications, researchers demonstrated that a detoxified derivative of SpA protected animals against intravenous infection with *S. aureus.* Mice and guinea pigs immunized with SpA and infected with *S. aureus*, if compared to only infected animals, mounted a broader antibody response against several *S. aureus* virulence factors otherwise masked by SpA activity. Finally, a possible positive impact that this unmasking effect could have against future infections was speculated (8, 41).

Based on these observations, a mouse model of skin infection and recurrence has been chosen to (i) evaluate whether immunization with SpA could unmask the pathogen also in a model of infection that resembled the most frequent staphylococcal disease in humans; (ii) measure the impact that this unmasking effect could have against a future encounter with bacteria in such a model.

Vaccination with SpA_mut_/AS01 did not directly protect animals against skin infections and related *sequelae* but triggered the host to mount a protective immune response during this first encounter, which proved to have a crucial importance for a second infection (**Figure 2**; see also **Supplementary Figure 1**). In fact, the immunization with the proposed vaccine contributed not only to increase the number of *S. aureus* virulence factors recognized during infection but also to improve the quality of the mounted immune response, which strongly limited the ability of *S. aureus* to use its elusive properties when reinfecting the host.

**Figure 6:**
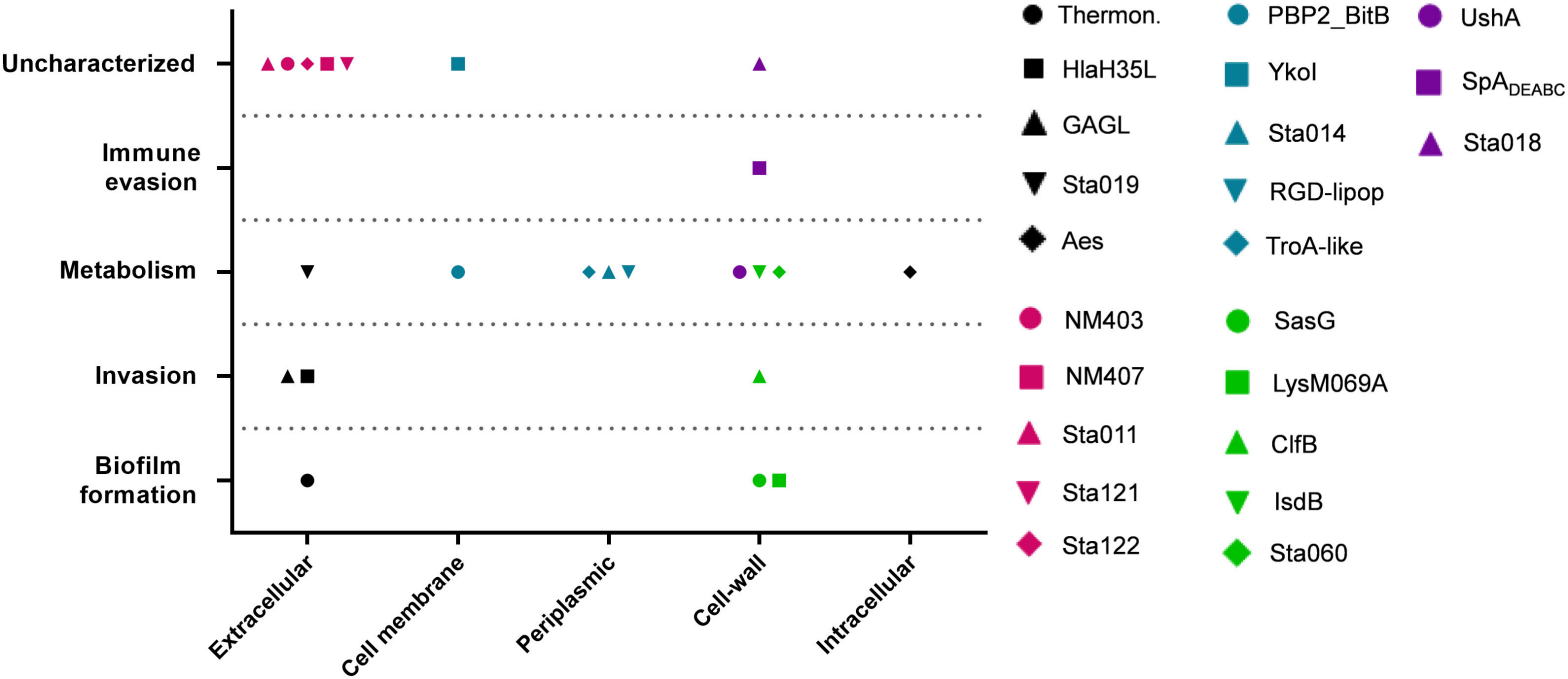
Correlation between cellular localization and functionality of recognized antigens. Graph summarizing cellular localization and pathogenic activity of the *S. aureus* virulence factors recognized by sera from SpA_mut_/AS01 immunized and *S. aureus*-infected mice. The 23 antigens recognized with higher frequency by sera from immunized and infected animals were further characterized by literature check and using the gene annotation present in NCBI data bank for their cellular localization (*X* axis) and *in vivo*/*in vitro* functionality (*Y* axis). Single dots reported information for single antigens as described in the legend. CSA family: conserved staphylococcal antigen family (32).

Despite being highly immunogenic, vaccination with SpA_mut_/AS01 had, *per se*, no or very poor effect against a primary *S. aureus* skin infection and related systemic complications (**Supplementary Figure 1**). Infection with the pathogen did not quantitatively boost the IgG response against SpA over the level obtained by immunization itself but, nevertheless, positively affected the quality of these antibodies (**Figure 1**). Anti-SpA antibodies in sera of vaccinated mice after a first encounter with *S. aureus* had an increased affinity for the target and showed higher efficacy in displacing IgGs bound by SpA, presumably also *in vivo* (**Figure 5**). Anti-SpA-specific IgG indeed confirmed to “undress” the pathogen during infection since sera from vaccinated and infected animals can recognize more antigens during infection as compared to sera from negative control animals (**Figure 4**; **Supplementary Table 2**). This increment was quantitatively relevant: while 20 out of 96 antigens assessed induced detectable antibodies during *S. aureus* infection in at least five out of nine sera tested, this number grew up to 53 in the presence of anti-SpA specific IgGs (Fisher’s exact test, *p*_val_< 0.0001). Localization of these antigens on the bacteria is very broad. Out of 23, 9 (39.1%) were secreted molecules, which are indeed more easily recognized by the immune system, followed by cell-wall-associated antigens (8 out of 23, 34.8%), antigens located in the periplasmic space (3 out of 23, 13%), molecules anchored to the cell membrane (2 out of 23, 8.7%), and, finally, cytoplasmic proteins (1 out of 23, 4.3%). Additionally, most of them are well-known virulence factors involved in biofilm formation or known to contribute to *S. aureus* invasiveness and colonization (**Table 2**, **Figure 6**). Finally, sera from immunized and infected animals showed the highest capacity to favor phagocytosis of bacteria by human monocytic cells *in vitro* as compared to sera of all the other groups (**Figure 3**). The impact of the general improvement of the IgG response against the pathogen resulted in significant reduction of bacterial load in the site of infection and, surprisingly, an almost complete eradication of bacteria in the kidneys used as a marker of systemic dissemination (**Figure 2**). Taken together, these data suggest that all these positive effects could play an important role against different pathogenic mechanisms, increasing the ability of the host to clear the infection. The occurrence of both these results, indeed, required the animals not only to be vaccinated with SpA, but also to be infected. Interestingly, a trend in CFU count reduction in skin homogenates was also observed in infected-only animals as compared to naïve mice, but this effect did not correlate with a systemic protection (**Figure 2**). Since alpha toxin (Hla) plays a central role for the development of dermonecrotic lesions and bacteria growth in the site of infection and since infection *per se* was sufficient to induce detectable anti-Hla antibodies, we might postulate that these antibodies might have played an important role to control skin infection (42, 43). On the other hand, the control of systemic *sequelae* probably needed a more diverse and complete immune response, like that developed by vaccinated and infected mice.

Interestingly, since human neutrophils utilize the same FcγRs to trigger phagocytosis, we could hypothesize that this mechanism of protection could also be extended in humans during a systemic spreading of *S. aureus*, even if *in vitro* phagocytosis performed with a human monocytic cell line could not directly correlate with a possible protection in vaccinated people (44–47).

To conclude, even if we could not exclude the idea that the cellular response may have had a partial contribution on the overall observed protection, IgG-mediated opsonophagocytosis is certainly one of the most important mechanisms of bacterial clearance that could explain our results (16, 48). These data strongly support the proposal to use this antigen in a vaccine against *S. aureus*-mediated SSTIs, and it would be better if it was combined with additional antigens that could offer direct protection during a first encounter with the pathogen.

## Data availability statement

The original contributions presented in the study are included in the article/**Supplementary Material**. Further inquiries can be directed to the corresponding author.

## Ethics statement

The animal study was approved by Italian Ministry of Health (authorization 123/2015-PR). The study was conducted in accordance with the local legislation and institutional requirements.

## Author contributions

AM: Data curation, Methodology, Writing – original draft, Writing – review & editing. GM: Data curation, Methodology, Writing – review & editing. MT: Methodology, Writing – review & editing. ST: Methodology, Writing – review & editing. DL: Writing – review & editing. FB: Writing – review & editing. OF: Writing – review & editing. GB: Writing – review & editing, Supervision. MB: Writing – review & editing, Supervision. EC: Data curation, Formal analysis, Methodology, Supervision, Writing – original draft, Writing – review & editing.
