# Supplementary material for: Vaccination with staphylococcal protein A protects mice against systemic complications of skin infection recurrences
Source: Front Immunol. 2024 Mar 11;15:1355764. doi: 10.3389/fimmu.2024.1355764 (PMC10961379; doi:10.3389/fimmu.2024.1355764)
Supplement: Supplementary file 1 [file DataSheet_1.docx]

Supplementary Material

# Figure S1

Figure S1. Experimental timeline of the mouse model of skin infection and recurrence. The model of skin infection and recurrence was performed in 5-week-old female C57BL/6N mice. At fixed timepoints (indicated with a red droplet) blood was collected from animals and sera were prepared as described in the relative material and methods section. IM: intramuscular; SC: subcute; organ collection: skin and kidneys. Created with BioRender.com by the author.


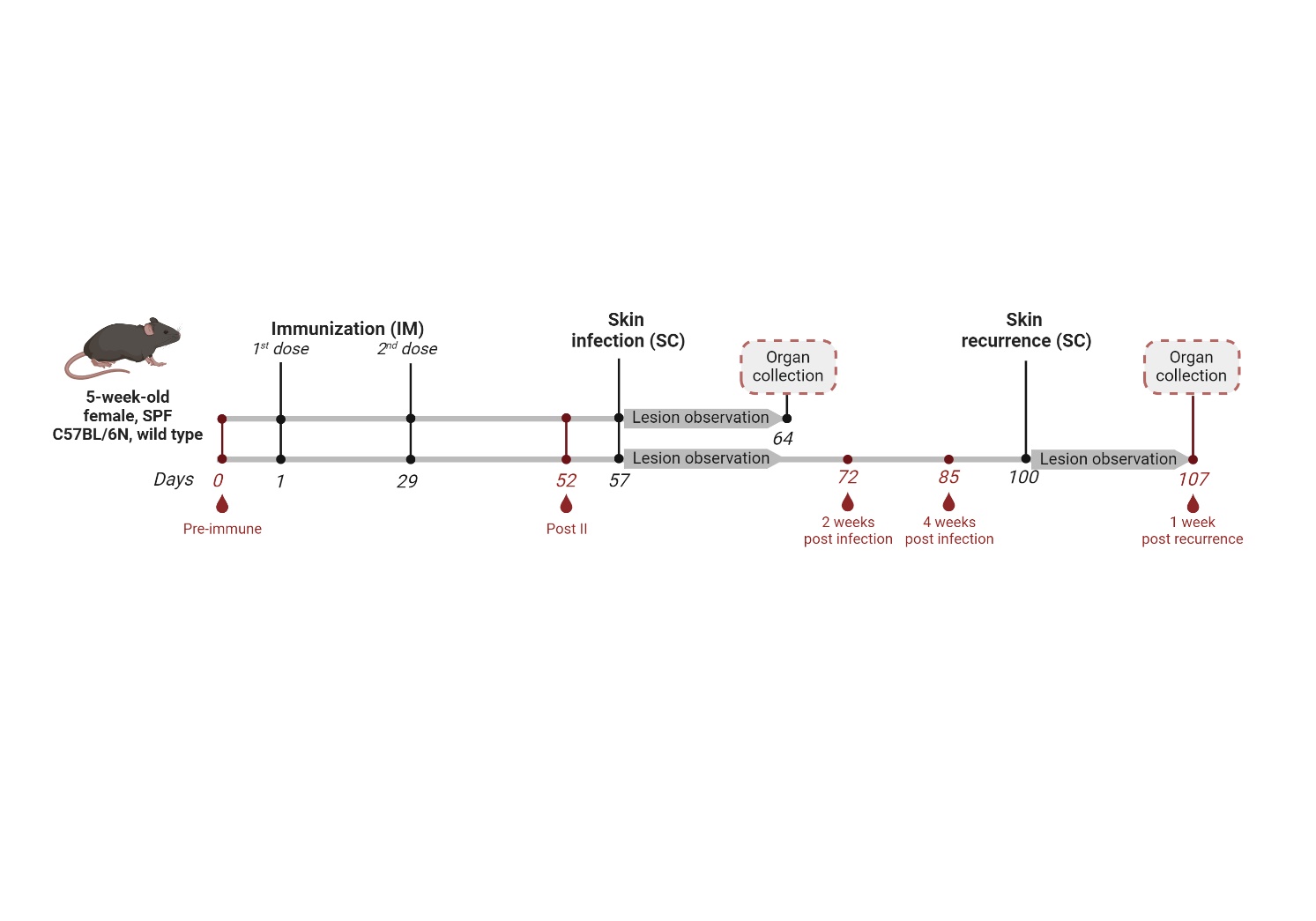


# Figure S2

Figure S2. SpA_mut_/AS01 immunization does not protect against skin infection. Skin (A and B) and systemic (C and D) readouts used to assess protection of SpA_mut_/AS01 vaccine in the model of skin infection. A) Area Under the Curve (AUC) expressed in mm^2^ of dermonecrotic lesion sizes developed from day four to day seven after skin infection (n=60 mice per group). Numbers above data from single groups represents the number of mice with detectable values vs the total number of mice/group. The red dotted line represents the Lower Detectable Value (LDV). B) Log_10_ of Colony Forming Units counts (CFUs) enumerated in homogenized skin biopsies collected fourteen days after skin infection (n=32 mice per group). The red dotted line represents the LDV. C) Dissemination severity index assigned to single mice based on CFU counts recovered in the kidneys of infected mice (n=10 mice per group). Score 0, no bacteria; Score 1, very low dissemination (1-10 CFU/kidneys); Score 2, minimal dissemination (11-100 CFU/kidneys); Score 3, mild dissemination (101-1000 CFU/kidneys); Score 4 severe dissemination (>1001 CFU/kidneys).

For all the graphs above, each single dot represents data from a single animal, red lines are median values of the groups. The Mann-Whitney t test was used to assess significance among groups.


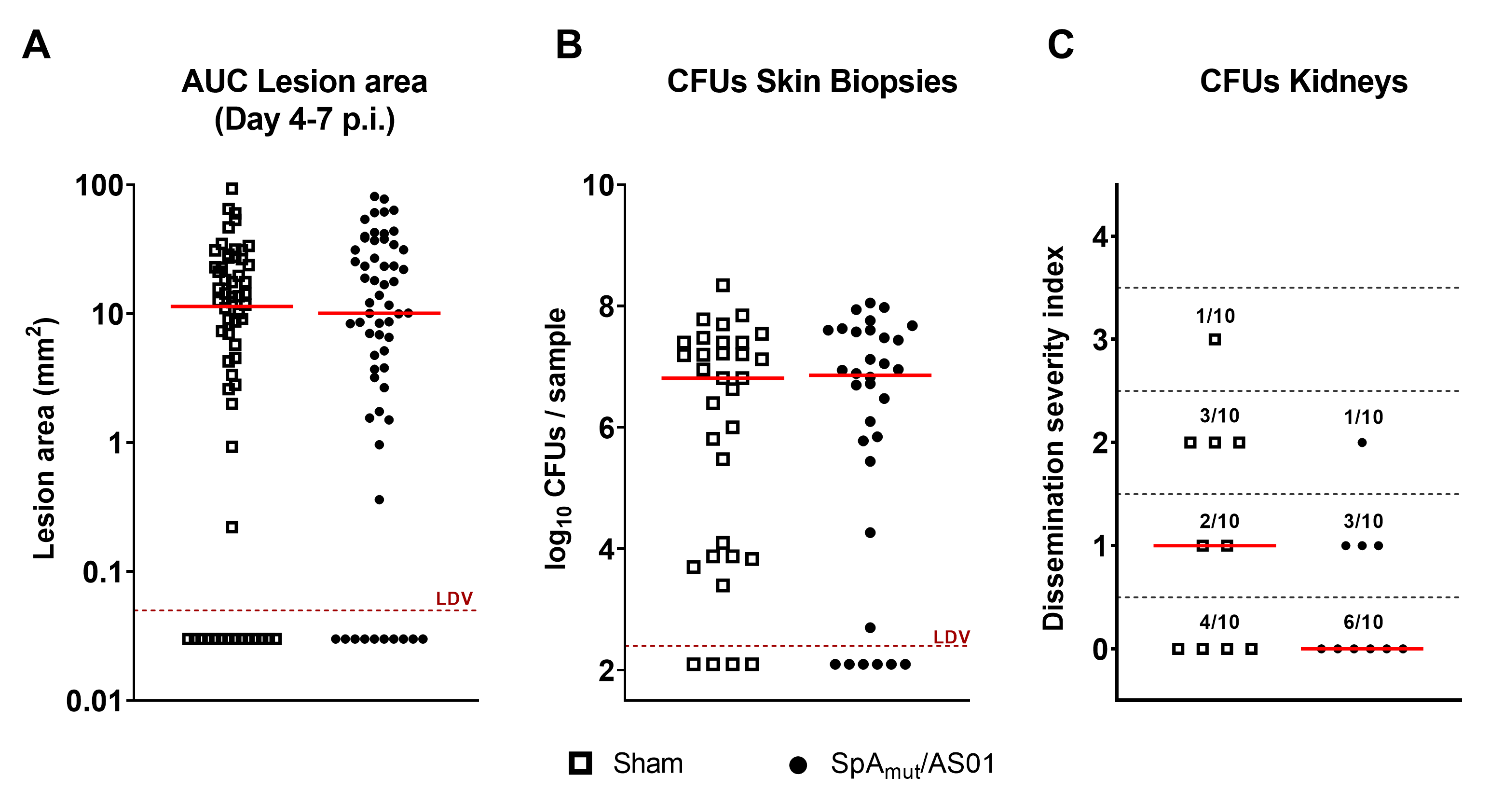


# Table ST1. Clinical scoresheet

|  | **Clinical Score Value** | | | | | | | |
| --- | --- | --- | --- | --- | --- | --- | --- | --- |
| Clinical symptoms | **0** | **0.5** | **1** | **1.5** | **2** | **2.5** | **3** | **4** |
| Scruffy coat |  | Mild | Moderate | Severe |  |  |  |  |
| Kyphosis |  |  | Mild | Moderate | Severe |  |  |  |
| Reduced deambulation |  |  |  | Mild | Moderate | Severe |  |  |
| Dermonecrotic lesion |  | Redness | Motile  Lesion | Non-motile  Lesion | Necrotic lesion – small | Necrotic lesion – medium | Necrotic lesion – large |  |
| Inactivity |  |  |  |  |  |  |  | Yes |
| Weight loss |  |  |  |  | 5% | 10% | 15% | 20% |

# Table ST2. Complete list of the staphylococcal antigens spotted on the protein chip microarray

| **Name** | **Description** | **Localization** | **N. reactive sera/N. Total sera tested** | |
| --- | --- | --- | --- | --- |
|  |  |  | **Immunized** | **Not immunized** |
| Aes | Acetyl esterase/lipase (Aes family) | Cytoplasmic | 9/9 | 2/9 |
| Autolysin fragment | Involved in biofilm formation | Cell-wall | 7/9 | 0/9 |
| Baa(petTEV) | Bacterial associated adhesin | Cell-wall | 1/9 | 0/9 |
| CHIPS | Chemotaxis-inhibitory protein of *S. aureus* | Extracellular | 6/9 | 4/9 |
| ClfA | Clumping factor A, binds to fibrinogen | Cell-wall | 8/9 | 4/9 |
| ClfB | Clumping factor B promotes colonization by binding fibrinogen, cytokeratin | Cell-wall | 9/9 | 7/9 |
| CoA | Staphylocoagulase | Extracellular | 3/9 | 1/9 |
| CP5-TT-tagless | Capsular polysaccharide type 5 | Capsule | 0/9 | 1/9 |
| CP8-TT-tagless | Capsular polysaccharide type 8 | Capsule | 0/9 | 0/9 |
| DltD | Integral membrane protein involved in the biosynthesis of D-alanyl-lipoteichoic acid | Cell membrane | 0/9 | 0/9 |
| Eap-GST | Extracellular adherence protein, interacts with several host cell matrix components, it has an anti-inflammatory and anti-angiogenic properties | Extracellular | 0/9 | 0/9 |
| EbpS (1-189) | Elastin binding protein | Cell membrane | 0/9 | 0/9 |
| EfbP | 5-nucleotidase family protein | Cell membrane | 7/9 | 5/9 |
| Emyy lipoprotein | Lipoprotein precursor, Emyy motif | Cell membrane | 9/9 | 8/9 |
| FeuA | Putative periplasmic binding protein, ABC transporter of metal ions | Periplasmic | 7/9 | 2/9 |
| FLIPr | Formyl peptide receptor-like 1 inhibitory protein, an anti-inflammatory protein that inhibits the leukocyte response to FPRL1 agonists | Extracellular | 2/9 | 1/9 |
| FnBA | Fibronectin binding protein A | Cell-wall | 9/9 | 7/9 |
| F-PVL | Component F of Pantone-Valentine toxin | Extracellular | 0/9 | 0/9 |
| FtsZ | Cell division protein that forms a contractile ring structure (Z ring) at the future cell division site | Cytoplasm | 0/9 | 0/9 |
| Glycosaminogl-ycan lyase | Member of lyase family, secreted enzymes able to act on glycosaminoglycans (hyaluronan and chondroitin), contributing to invasive capacity of *S. aureus* | Extracellular | 7/9 | 0/9 |
| Hla H35L | α-haemolysin, cytotoxic | Extracellular | 9/9 | 3/9 |
| HlgA | γ-hemolysin component A | Extracellular | 5/9 | 1/9 |
| HlgC | γ-hemolysin component C, leukocidin | Extracellular | 7/9 | 2/9 |
| IsdA | Iron-regulated surface determinant protein A, heme binding domain | Cell-wall | 6/9 | 2/9 |
| IsdB | Iron-regulated surface determinant protein B, heme binding domain | Cell-wall | 7/9 | 1/9 |
| IsdE | ABC transporter-binding protein | Cell membrane | 0/9 | 0/9 |
| IsdI | heme-degrading enzyme, monooxygenase | Extracellular | 0/9 | 0/9 |
| IsaA | Immunodominant staphylococcal antigen A | Cell-wall, secreted | 0/9 | 0/9 |
| IsspA | Intracellular serine protease - Peptidase S8 domain Lantiobiotic (lanthionine-containing antibiotics) specific proteases | Cytoplasm | 9/9 | 8/9 |
| LukE | Leucotoxin LukE | Extracellular | 0/9 | 0/9 |
| MdoB | Phosphoglycerol transferase MdoB | Cell membrane | 3/9 | 0/9 |
| nasD | NAD(P)/FAD-dependent oxidoreductase | Cytoplasm | 3/9 | 0/9 |
| NM011 | Uncharacterized staphylococcal antigen belonging to CSA family | Extracellular | 8/9 | 0/9 |
| NM026 | Uncharacterized staphylococcal antigen belonging to CSA family | Extracellular | 9/9 | 9/9 |
| NM066 | Uncharacterized staphylococcal antigen belonging to CSA family | Extracellular | 1/9 | 1/9 |
| NM121 | Uncharacterized staphylococcal lipoprotein belonging to CSA family | Extracellular | 7/9 | 1/9 |
| NM122 | Uncharacterized staphylococcal lipoprotein belonging to CSA family | Extracellular | 6/9 | 0/9 |
| NM123 | Uncharacterized staphylococcal antigen belonging to CSA family | Extracellular | 8/9 | 6/9 |
| NM409 | (Paralog) uncharacterized staphylococcal antigen belonging to CSA family | Extracellular | 3/9 | 0/9 |
| NM411 | (Paralog) uncharacterized staphylococcal antigen belonging to CSA family | Extracellular | 2/9 | 0/9 |
| NM403 | (Paralog) uncharacterized staphylococcal antigen belonging to CSA family | Extracellular | 8/9 | 3/9 |
| NM405 | (Paralog) uncharacterized staphylococcal antigen belonging to CSA family | Extracellular | 0/9 | 0/9 |
| NM406 | (Paralog) uncharacterized staphylococcal antigen belonging to CSA family | Extracellular | 0/9 | 0/9 |
| NM407 | (Paralog) uncharacterized staphylococcal antigen belonging to CSA family | Extracellular | 5/9 | 0/9 |
| NM408 | (Paralog) uncharacterized staphylococcal antigen belonging to CSA family | Extracellular | 8/9 | 4/9 |
| NW_2 | DUF2798 domain-containing protein | ND | 2/9 | 0/9 |
| PBP2_BitB | Substrate binding domain of a putative iron transporter BitB, member of the PBP2 family. | Cell membrane | 9/9 | 3/9 |
| PBP2_NrtA_CpmA-like protein | Member of the PBP2 superfamily, binding domain of nitrate transporter | Periplasmic | 9/9 | 1/9 |
| Peptide ABC transporter | Member of the OppA transport system, substrate-binding protein | Cell-wall | 9/9 | 3/9 |
| Peptidoglycan hydrolase | Petidoglycan hydrolase | Extracellular | 9/9 | 1/9 |
| pEstA | EstA lipase precursor | ND | 9/9 | 8/9 |
| prsA | Foldase protein, helps the post-translocational extracellular folding of several secreted proteins | Cell membrane | 0/9 | 0/9 |
| RGD-containing lipoprotein | Member of the OppA transport system, plays a role in recycling cell wall peptides | Periplasmic | 9/9 | 0/9 |
| SabG | 1-phosphatidylinositol phosphodiesterase | ND | 3/9 | 4/9 |
| SasD | Surface protein SasD | Cell-wall | 0/9 | 1/9 |
| SasG (53-608) | Accumulation protein, biofilms | Cell-wall | 7/9 | 1/9 |
| Sbi | Immunoglobulin-binding protein Sbi | Cell-wall, secreted | 0/9 | 0/9 |
| SdrC | fibrinogen-binding protein SdrC | Cell-wall | 5/9 | 2/9 |
| SdrD | fibrinogen-binding protein SdrD | Cell-wall | 9/9 | 8/9 |
| SdrE | MSCRAMM family adhesin SdrE | Cell-wall, secreted | 9/9 | 9/9 |
| selX | ssl-like Enterotoxin-like toxin X, plays a role in the inhibition of the host innate immune system. Inhibits phagocytosis and killing by human neutrophils | Extracellular | 8/9 | 6/9 |
| SpA-KKAA | Staphyloccocal protein A - detoxified | Cell-wall | 9/9 | 2/9 |
| SpA-WT | Staphyloccocal protein A | Cell-wall | 0/9 | 0/9 |
| Sph | Sphingomyelin phosphodiesterase | Extracellular | 9/9 | 8/9 |
| SplB | Serine protease | Extracellular | 6/9 | 6/9 |
| SplC | Serine protease | Extracellular | 5/9 | 5/9 |
| SsaA-like | secretory antigen SsaA-like protein | Extracellular | 0/9 | 0/9 |
| ssl11 | Staphylococcal superantigen-like 11, induces proinflammatory cytokines from peripheral blood mononuclear cells | Extracellular | 3/9 | 1/9 |
| ssl3 | Staphylococcal superantigen-like 3, plays an essential role in immune innate response inhibition by interacting with and inhibiting host TLR2 | Extracellular | 8/9 | 4/9 |
| ssl4 | Staphylococcal superantigen-like 4, plays a role in immune innate response inhibition by interfering with host TLR2-mediated pathway. | Extracellular | 8/9 | 6/9 |
| ssl5 | Staphylococcal superantigen-like 5, plays a role in the inhibition of host innate immune system by preventing initial rolling of neutrophils toward the site of infection. | Extracellular | 5/9 | 4/9 |
| ssl6 | Staphylococcal superantigen-like 6, interacts with neutrophils receptors | Extracellular | 0/9 | 0/9 |
| ssl7 | Staphylococcal superantigen-like 7, plays a role in the inhibition of host complement-mediated lysis and serum bactericidal activity by interacting with complement component C5 | Extracellular | 3/9 | 0/9 |
| ssl8 | Staphylococcal superantigen-like 8, binds to tenascin C (TNC), a glycoprotein and inhibits TNC and fibronectin interaction and cell motility in keratinocytes | Extracellular | 3/9 | 3/9 |
| Sta018 | Substrate-binding component of an uncharacterized ABC-type nickel/dipeptide/oligopeptide-like import system | Cell-wall | 5/9 | 0/9 |
| Sta021 | CHAP-domaning containing protein | ND | 8/9 | 4/9 |
| Sta022 GST | MHC class II analog protein | Cell membrane | 9/9 | 9/9 |
| Sta030 | Hypothetical protein | ND | 0/9 | 0/9 |
| Sta042 | Hypothetical protein | ND | 6/9 | 2/9 |
| Sta051 | Putative adhesin | ND | 3/9 | 1/9 |
| Sta108 | Hypothetical protein, DM-13 domain-containing protein | ND | 9/9 | 9/9 |
| Sta111 | Hypothetical protein, contains domain of unknown function (DUF5011) | ND | 1/9 | 0/9 |
| Sta115 | Uncharacterized lipoprotein containing domain of unknown function with cystatin-like fold (DUF4467) | Cell membrane | 4/9 | 0/9 |
| Thermonuclease homologous | Regulates biofilm formation by modulating extracellular DNA release | Extracellular | 9/9 | 0/9 |
| TroA-like transporter | TroA-like superfamily trasport ferric siderophores and metal ions | Periplasmic | 0/9 | 0/9 |
| TroA-like transporter | TroA-like superfamily trasport ferric siderophores and metal ions | Periplasmic | 4/9 | 2/9 |
| TroA-like transporter | TroA-like superfamily trasport ferric siderophores and metal ions | Periplasmic | 5/9 | 0/9 |
| TroA-like transporter | TroA-like superfamily trasport ferric siderophores and metal ions | Periplasmic | 0/9 | 0/9 |
| TroA-like transporter | TroA-like superfamily trasport ferric siderophores and metal ions | Periplasmic | 9/9 | 9/9 |
| TroA-like transporter | TroA-like superfamily trasport ferric siderophores and metal ions | Periplasmic | 9/9 | 9/9 |
| Tetanus toxoid (TT) | Carrier for CP5 and CP8 | N/A | 9/9 | 5/9 |
| UshA | Belonging to 5' nucleotidase family | Cell-wall | 9/9 | 0/9 |
| VWbp | von Willebrand factor binding protein | Extracellular | 3/9 | 0/9 |
| YkoI | Uncharacterized membrane protein (Ykoi) | Cell membrane | 8/9 | 3/9 |

ND: not determined; N/A: not applicable. If not indicated otherwise (tagless or GST – gluthatione S-H transferase, TT – tetanus toxoid carrier), the antigen is to be considered 6X His-tagged
